# Bis{(*E*)-4-chloro-2-[(2-chloro-3-pyrid­yl)imino­methyl-κ*N*]phenolato-κ*O*}copper(II)

**DOI:** 10.1107/S1600536809029651

**Published:** 2009-07-31

**Authors:** Yu-Jie Ding, Jun-Feng Tong, Wen-Kui Dong, Yin-Xia Sun, Jian Yao

**Affiliations:** a Department of Biochemical Engineering, Anhui University of Technology and Science, Wuhu 241000, People’s Republic of China; b School of Chemical and Biological Engineering, Lanzhou Jiaotong University, Lanzhou 730070, People’s Republic of China

## Abstract

In the title complex, [Cu(C_12_H_7_Cl_2_N_2_O)_2_], the Cu^II^ center is tetra­coordinated by two phenolic O and two azomethine N atoms from two bidentate 4-chloro-2-[(2-chloro-3-pyrid­yl)imino­meth­yl]phenolate (*L*) ligands. In the crystal structure, the Cu^II^ atom has a distorted square-planar coordination environment. The dihedral angles between the benzene and pyridyl rings are 54.39 (3) and 80.14 (4)°, indicating that the pyridine ring has a considerably weaker steric hindrance. The packing of the mol­ecule is controlled by C—H⋯π(Ph) inter­actions and short O⋯Cl inter­actions [3.196 (4) Å], linking the mol­ecules into a chain-like structure along the *c* axis.

## Related literature

For background to Schiff bases, see: Soliman & Mohamed (2004 ▶); Abd El-Wahab *et al.* (2004 ▶). For the synthesis, see: Dong *et al.* (2009*d* ▶). For related structures, see: Dong *et al.* (2009**a* ▶,*b* ▶,c* ▶).
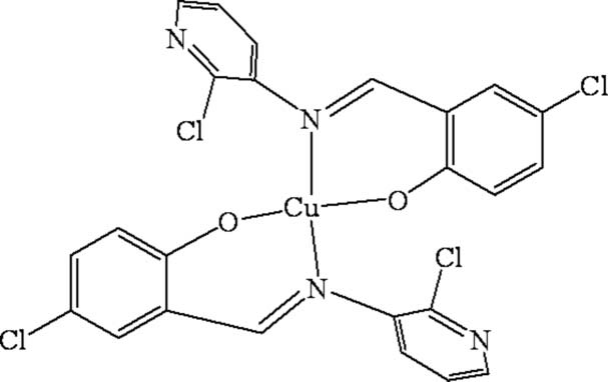


## Experimental

### 

#### Crystal data

[Cu(C_12_H_7_Cl_2_N_2_O)_2_]
*M* *_r_* = 595.73
Monoclinic, 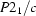

*a* = 20.236 (2) Å
*b* = 11.4821 (14) Å
*c* = 10.5458 (9) Å
β = 90.132 (2)°
*V* = 2450.3 (4) Å^3^
*Z* = 4
Mo *K*α radiation
μ = 1.36 mm^−1^
*T* = 298 K
0.40 × 0.14 × 0.09 mm

#### Data collection

Buker SMART 1000 CCD area-detector diffractometer
Absorption correction: multi-scan (*SADABS*; Sheldrick, 1996 ▶) *T*_min_ = 0.613, *T*_max_ = 0.888
12294 measured reflections
4320 independent reflections
2614 reflections with *I* > 2σ(*I*)
*R*_int_ = 0.058

#### Refinement

*R*[*F* ^2^ > 2σ(*F* ^2^)] = 0.043
*wR*(*F* ^2^) = 0.065
*S* = 1.02
4320 reflections
316 parameters
H-atom parameters constrained
Δρ_max_ = 0.42 e Å^−3^
Δρ_min_ = −0.39 e Å^−3^

### 

Data collection: *SMART* (Siemens, 1996 ▶); cell refinement: *SAINT* (Siemens, 1996 ▶); data reduction: *SAINT*; program(s) used to solve structure: *SHELXS97* (Sheldrick, 2008 ▶); program(s) used to refine structure: *SHELXL97* (Sheldrick, 2008 ▶); molecular graphics: *SHELXTL* (Sheldrick, 2008 ▶); software used to prepare material for publication: *SHELXTL*.

## Supplementary Material

Crystal structure: contains datablocks global, I. DOI: 10.1107/S1600536809029651/at2850sup1.cif

Structure factors: contains datablocks I. DOI: 10.1107/S1600536809029651/at2850Isup2.hkl

Additional supplementary materials:  crystallographic information; 3D view; checkCIF report

## Figures and Tables

**Table 1:** Hydrogen-bond geometry (Å, °)

*D*—H⋯*A*	*D*—H	H⋯*A*	*D*⋯*A*	*D*—H⋯*A*
C22—H22⋯*Cg*1^i^	0.93	2.89	3.753 (3)	155

Symmetry code: (i) 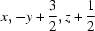. *Cg*1 is the centroid of the C2–C7 ring.

**Fig. 1.**
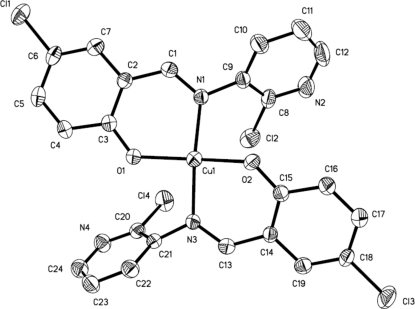
The molecular structure of the title complex with atom numbering scheme. Displacement ellipsoids for non-hydrogen atoms are drawn at the 30% probability level.

**Fig. 2.**
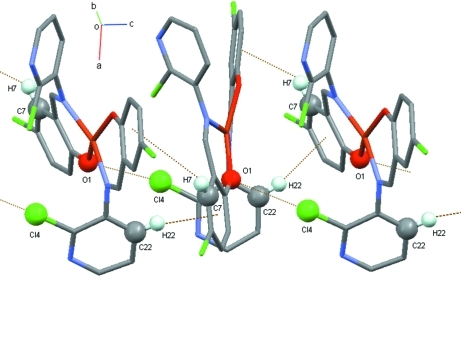
The infinite one-dimensional supramolecular structure along c axis linked by C—H···π(Ph) interactions and short O···Cl interactions (dashed lines).

## supplementary crystallographic information

### Comment

Schiff bases are a versatile class of ligands in the field of modern coordination chemistry (Soliman & Mohamed, 2004), which can coordinate to transition or rare earth ions yielding complexes with interesting properties that are useful in materials science and in biological systems (Abd El-Wahab *et al.*, 2004). As an extension of our work on the complexes between transition metals and Schiff base ligands (Dong *et al.*, 2009*a*; Dong *et al.*, 2009*b*), we report here the synthesis and crystal structures of the title complex, bis{(*E*)-[4-chloro-2-((2-chloropyridin-3-ylimino)methyl-κ*N*)] phenolato-κ*O*^1^}copper(II) (Fig. 1).

In asymmetric molecule unit of the title complex, the Cu^II^ center is tetracoordinated by two phenolic O and two azomethine N atoms from two ligand (*L*^-^) units and has a distorted square-planar coordination environment, which is similar to the reported copper complex with (*E*)-[4-bromo-2-((2-chloropyridin-3-ylimino)methyl)]phenol (Dong *et al.*, 2009*c*). The interplane dihedral angles are found to be as follows: 54.39 (3)° between the benzene ring (C2—C7) and pyridyl ring (N2/C8—C12), 80.14 (4)° between benzene ring (C14—C19) and pyridyl ring (N4/C20—C24), indicating the pyridine ring having a considerable weaker steric hindrance. Besides, the dihedral angle between the coordination plane of O1—Cu1—N1 and O2—Cu1—N3 is 27.96 (3)°, indicating slight distortion toward tetrahedral geometry from the square planar structure. The packing of the molecule is controlled by C—H···π(Ph) interactions and short O···Cl interactions linking molecules into infinite one-dimensional supramolecular structure along *c* axis (Fig. 2).

### Experimental

(*E*)-[4-Chloro-2-((2-chloropyridin-3-ylimino)methyl)]phenol (HL) was prepared according to previously reported procedure (Dong *et al.*, 2009d). A blue solution of copper(II) acetate monohydrate (4.2 mg, 0.021 mmol) in ethanol (2 ml) was added dropwise to a pale-yellow solution of HL (11.2 mg, 0.042 mmol) in ethanol (4 ml) at room temperature. The colour of the mixing solution turned to brown immediately, then allowed to stand at room temperature for several days. With evaporation of the solvent, brown needle-like single crystals suitable for X-ray crystallographic analysis were obtained. IR: ν C=N, 1610 cm^-1^, ν Ar—O, 1236 cm^-1^, ν Cu—N, 459 cm^-1^ and ν Cu—O, 426 cm^-1^.

### Refinement

H atoms were treated as riding atoms with distances C—H = 0.93 Å (CH), and *U*_iso_(H) = 1.2U_eq_(C).

### Crystal data

**Table:**

[Cu(C_12_H_7_Cl_2_N_2_O)_2_]	*F*(000) = 1196
*M**_r_* = 595.73	*D*_x_ = 1.615 Mg m^−^^3^
Monoclinic, *P*2_1_/*c*	Mo *K*α radiation, λ = 0.71073 Å
Hall symbol: -P 2ybc	Cell parameters from 2435 reflections
*a* = 20.236 (2) Å	θ = 26.2–25.3°
*b* = 11.4821 (14) Å	µ = 1.36 mm^−^^1^
*c* = 10.5458 (9) Å	*T* = 298 K
β = 90.132 (2)°	Needle-like, brown
*V* = 2450.3 (4) Å^3^	0.40 × 0.14 × 0.09 mm
*Z* = 4	

### Data collection

**Table:**

Buker SMART 1000 CCD area-detector diffractometer	4320 independent reflections
Radiation source: fine-focus sealed tube	2614 reflections with *I* > 2σ(*I*)
graphite	*R*_int_ = 0.058
φ and ω scans	θ_max_ = 25.0°, θ_min_ = 2.0°
Absorption correction: multi-scan (*SADABS*; Sheldrick, 1996)	*h* = −24→19
*T*_min_ = 0.613, *T*_max_ = 0.888	*k* = −13→13
12294 measured reflections	*l* = −12→12

### Refinement

**Table:**

Refinement on *F*^2^	Primary atom site location: structure-invariant direct methods
Least-squares matrix: full	Secondary atom site location: difference Fourier map
*R*[*F*^2^ > 2σ(*F*^2^)] = 0.043	Hydrogen site location: inferred from neighbouring sites
*wR*(*F*^2^) = 0.065	H-atom parameters constrained
*S* = 1.01	*w* = 1/[σ^2^(*F*_o_^2^) + (0.0103*P*)^2^] where *P* = (*F*_o_^2^ + 2*F*_c_^2^)/3
4320 reflections	(Δ/σ)_max_ = 0.001
316 parameters	Δρ_max_ = 0.42 e Å^−^^3^
0 restraints	Δρ_min_ = −0.39 e Å^−^^3^

### Special details

**Table:**

Geometry. All e.s.d.'s (except the e.s.d. in the dihedral angle between two l.s. planes) are estimated using the full covariance matrix. The cell e.s.d.'s are taken into account individually in the estimation of e.s.d.'s in distances, angles and torsion angles; correlations between e.s.d.'s in cell parameters are only used when they are defined by crystal symmetry. An approximate (isotropic) treatment of cell e.s.d.'s is used for estimating e.s.d.'s involving l.s. planes.
Refinement. Refinement of *F*^2^ against ALL reflections. The weighted *R*-factor *wR* and goodness of fit *S* are based on *F*^2^, conventional *R*-factors *R* are based on *F*, with *F* set to zero for negative *F*^2^. The threshold expression of *F*^2^ > σ(*F*^2^) is used only for calculating *R*-factors(gt) *etc*. and is not relevant to the choice of reflections for refinement. *R*-factors based on *F*^2^ are statistically about twice as large as those based on *F*, and *R*- factors based on ALL data will be even larger.

### Fractional atomic coordinates and isotropic or equivalent isotropic displacement parameters (Å^2^)

**Table:**

	*x*	*y*	*z*	*U*_iso_*/*U*_eq_	
Cu1	0.75122 (2)	0.77649 (3)	0.42109 (4)	0.04479 (14)	
Cl1	0.85318 (5)	0.19528 (7)	0.24296 (11)	0.0748 (4)	
Cl2	0.69809 (5)	0.87831 (8)	0.16425 (10)	0.0715 (3)	
Cl3	0.63434 (5)	1.36121 (8)	0.53290 (13)	0.0847 (4)	
Cl4	0.87717 (5)	0.87627 (9)	0.20907 (10)	0.0712 (3)	
N1	0.69574 (13)	0.6664 (2)	0.3246 (3)	0.0404 (7)	
N2	0.5723 (2)	0.8484 (3)	0.1640 (4)	0.0824 (12)	
N3	0.81090 (13)	0.9095 (2)	0.4590 (3)	0.0405 (7)	
N4	0.97911 (16)	0.8403 (3)	0.3522 (3)	0.0580 (9)	
O1	0.82307 (10)	0.67264 (18)	0.4291 (2)	0.0496 (7)	
O2	0.67527 (11)	0.85984 (17)	0.4727 (2)	0.0475 (7)	
C1	0.71125 (17)	0.5582 (3)	0.3021 (3)	0.0434 (9)	
H1	0.6795	0.5116	0.2634	0.052*	
C2	0.77345 (17)	0.5055 (3)	0.3324 (3)	0.0402 (9)	
C3	0.82696 (17)	0.5658 (3)	0.3882 (3)	0.0387 (9)	
C4	0.88764 (17)	0.5059 (3)	0.3980 (3)	0.0493 (10)	
H4	0.9234	0.5434	0.4355	0.059*	
C5	0.89566 (19)	0.3951 (3)	0.3546 (4)	0.0515 (10)	
H5	0.9366	0.3587	0.3612	0.062*	
C6	0.8432 (2)	0.3370 (3)	0.3011 (4)	0.0507 (11)	
C7	0.78289 (18)	0.3887 (3)	0.2908 (3)	0.0479 (10)	
H7	0.7476	0.3474	0.2564	0.057*	
C8	0.62746 (19)	0.8039 (3)	0.2075 (4)	0.0560 (11)	
C9	0.63246 (18)	0.7033 (3)	0.2809 (3)	0.0459 (10)	
C10	0.5749 (2)	0.6457 (3)	0.3112 (4)	0.0635 (12)	
H10	0.5757	0.5791	0.3614	0.076*	
C11	0.5154 (2)	0.6894 (4)	0.2649 (5)	0.0825 (15)	
H11	0.4756	0.6519	0.2810	0.099*	
C12	0.5177 (3)	0.7896 (5)	0.1948 (5)	0.0967 (18)	
H12	0.4777	0.8195	0.1661	0.116*	
C13	0.79004 (17)	1.0141 (3)	0.4799 (3)	0.0432 (9)	
H13	0.8221	1.0711	0.4922	0.052*	
C14	0.72224 (17)	1.0512 (3)	0.4859 (3)	0.0398 (9)	
C15	0.66883 (18)	0.9718 (3)	0.4849 (3)	0.0421 (9)	
C16	0.60489 (17)	1.0190 (3)	0.5003 (3)	0.0522 (11)	
H16	0.5688	0.9689	0.5007	0.063*	
C17	0.59427 (18)	1.1356 (3)	0.5146 (4)	0.0575 (11)	
H17	0.5515	1.1639	0.5245	0.069*	
C18	0.64736 (19)	1.2118 (3)	0.5142 (4)	0.0531 (11)	
C19	0.71064 (18)	1.1714 (3)	0.5010 (3)	0.0489 (10)	
H19	0.7459	1.2233	0.5021	0.059*	
C20	0.91648 (19)	0.8681 (3)	0.3548 (4)	0.0453 (10)	
C21	0.88043 (17)	0.8917 (3)	0.4636 (4)	0.0403 (9)	
C22	0.91409 (18)	0.8877 (3)	0.5758 (4)	0.0525 (10)	
H22	0.8923	0.9029	0.6516	0.063*	
C23	0.98051 (19)	0.8610 (3)	0.5768 (4)	0.0582 (11)	
H23	1.0044	0.8594	0.6522	0.070*	
C24	1.01031 (19)	0.8367 (3)	0.4626 (5)	0.0594 (12)	
H24	1.0548	0.8166	0.4631	0.071*	

### Atomic displacement parameters (Å^2^)

**Table:**

	*U*^11^	*U*^22^	*U*^33^	*U*^12^	*U*^13^	*U*^23^
Cu1	0.0435 (3)	0.0421 (2)	0.0487 (3)	0.0027 (2)	−0.0027 (2)	−0.0051 (3)
Cl1	0.0885 (8)	0.0468 (6)	0.0891 (9)	0.0160 (6)	0.0088 (7)	−0.0087 (6)
Cl2	0.0949 (9)	0.0575 (6)	0.0622 (8)	0.0006 (6)	−0.0049 (6)	0.0104 (6)
Cl3	0.0771 (8)	0.0416 (5)	0.1354 (12)	0.0083 (5)	0.0144 (8)	−0.0066 (7)
Cl4	0.0742 (8)	0.0955 (7)	0.0439 (7)	0.0157 (6)	0.0002 (6)	−0.0041 (6)
N1	0.0402 (19)	0.0404 (17)	0.041 (2)	0.0038 (14)	−0.0034 (15)	−0.0013 (15)
N2	0.077 (3)	0.082 (3)	0.088 (3)	0.029 (2)	−0.037 (3)	−0.017 (2)
N3	0.0379 (19)	0.0411 (16)	0.042 (2)	0.0045 (14)	−0.0024 (15)	−0.0052 (15)
N4	0.047 (2)	0.067 (2)	0.060 (3)	0.0051 (18)	0.0065 (19)	−0.003 (2)
O1	0.0427 (15)	0.0416 (13)	0.0644 (19)	0.0052 (12)	−0.0101 (13)	−0.0088 (13)
O2	0.0440 (15)	0.0368 (13)	0.0616 (19)	0.0004 (12)	0.0039 (13)	−0.0083 (13)
C1	0.042 (2)	0.047 (2)	0.041 (3)	−0.0048 (19)	−0.0006 (19)	0.0013 (19)
C2	0.042 (2)	0.040 (2)	0.038 (2)	0.0023 (18)	0.0029 (19)	−0.0009 (19)
C3	0.038 (2)	0.044 (2)	0.035 (2)	0.0022 (18)	0.0044 (18)	0.0038 (19)
C4	0.041 (2)	0.056 (2)	0.051 (3)	0.006 (2)	−0.0053 (19)	0.002 (2)
C5	0.052 (3)	0.050 (2)	0.052 (3)	0.017 (2)	0.006 (2)	0.007 (2)
C6	0.062 (3)	0.040 (2)	0.050 (3)	0.014 (2)	0.008 (2)	0.000 (2)
C7	0.058 (3)	0.045 (2)	0.041 (3)	0.000 (2)	0.002 (2)	0.0018 (19)
C8	0.058 (3)	0.057 (3)	0.053 (3)	0.021 (2)	−0.010 (2)	−0.012 (2)
C9	0.044 (3)	0.052 (2)	0.042 (3)	0.010 (2)	−0.006 (2)	−0.011 (2)
C10	0.047 (3)	0.074 (3)	0.069 (3)	0.004 (2)	−0.003 (2)	−0.018 (2)
C11	0.047 (3)	0.107 (4)	0.094 (4)	0.001 (3)	−0.004 (3)	−0.031 (3)
C12	0.060 (4)	0.120 (5)	0.109 (5)	0.040 (4)	−0.035 (3)	−0.038 (4)
C13	0.044 (2)	0.045 (2)	0.041 (3)	−0.0035 (19)	0.0015 (19)	0.0000 (19)
C14	0.038 (2)	0.041 (2)	0.040 (2)	0.0049 (18)	0.0003 (18)	−0.0015 (18)
C15	0.040 (2)	0.048 (2)	0.038 (2)	0.0036 (19)	−0.0011 (18)	−0.0047 (19)
C16	0.041 (2)	0.048 (2)	0.067 (3)	−0.0001 (19)	0.002 (2)	−0.001 (2)
C17	0.046 (3)	0.053 (2)	0.074 (3)	0.006 (2)	−0.001 (2)	−0.004 (2)
C18	0.050 (3)	0.035 (2)	0.074 (3)	0.008 (2)	0.003 (2)	−0.006 (2)
C19	0.049 (2)	0.042 (2)	0.056 (3)	−0.0046 (19)	−0.001 (2)	0.003 (2)
C20	0.050 (3)	0.043 (2)	0.043 (3)	0.0017 (19)	0.001 (2)	−0.0012 (19)
C21	0.042 (2)	0.036 (2)	0.043 (3)	−0.0001 (18)	−0.001 (2)	−0.0007 (19)
C22	0.053 (3)	0.056 (2)	0.048 (3)	0.000 (2)	−0.002 (2)	0.000 (2)
C23	0.050 (3)	0.061 (2)	0.063 (3)	−0.003 (2)	−0.014 (2)	0.007 (2)
C24	0.044 (3)	0.057 (3)	0.077 (4)	−0.002 (2)	0.004 (3)	0.005 (3)

### Geometric parameters (Å, °)

**Table:**

Cu1—O1	1.882 (2)	C6—C7	1.361 (4)
Cu1—O2	1.892 (2)	C7—H7	0.9300
Cu1—N1	1.972 (3)	C8—C9	1.394 (5)
Cu1—N3	1.987 (3)	C9—C10	1.379 (4)
Cl1—C6	1.751 (3)	C10—C11	1.391 (5)
Cl2—C8	1.727 (4)	C10—H10	0.9300
Cl3—C18	1.747 (3)	C11—C12	1.368 (6)
Cl4—C20	1.731 (4)	C11—H11	0.9300
N1—C1	1.303 (3)	C12—H12	0.9300
N1—C9	1.424 (4)	C13—C14	1.438 (4)
N2—C8	1.309 (4)	C13—H13	0.9300
N2—C12	1.336 (5)	C14—C19	1.409 (4)
N3—C13	1.292 (3)	C14—C15	1.414 (4)
N3—C21	1.422 (4)	C15—C16	1.413 (4)
N4—C20	1.307 (4)	C16—C17	1.365 (4)
N4—C24	1.324 (5)	C16—H16	0.9300
O1—C3	1.303 (3)	C17—C18	1.385 (4)
O2—C15	1.298 (3)	C17—H17	0.9300
C1—C2	1.432 (4)	C18—C19	1.369 (4)
C1—H1	0.9300	C19—H19	0.9300
C2—C3	1.413 (4)	C20—C21	1.388 (4)
C2—C7	1.423 (4)	C21—C22	1.365 (5)
C3—C4	1.411 (4)	C22—C23	1.379 (4)
C4—C5	1.362 (4)	C22—H22	0.9300
C4—H4	0.9300	C23—C24	1.376 (5)
C5—C6	1.374 (5)	C23—H23	0.9300
C5—H5	0.9300	C24—H24	0.9300
			
O1—Cu1—O2	159.31 (10)	C9—C10—H10	120.8
O1—Cu1—N1	93.20 (11)	C11—C10—H10	120.8
O2—Cu1—N1	90.63 (10)	C12—C11—C10	117.6 (5)
O1—Cu1—N3	90.50 (10)	C12—C11—H11	121.2
O2—Cu1—N3	92.70 (10)	C10—C11—H11	121.2
N1—Cu1—N3	160.32 (11)	N2—C12—C11	125.8 (5)
C1—N1—C9	116.2 (3)	N2—C12—H12	117.1
C1—N1—Cu1	124.6 (3)	C11—C12—H12	117.1
C9—N1—Cu1	119.1 (2)	N3—C13—C14	126.5 (3)
C8—N2—C12	115.0 (4)	N3—C13—H13	116.7
C13—N3—C21	116.8 (3)	C14—C13—H13	116.7
C13—N3—Cu1	123.4 (2)	C19—C14—C15	120.3 (3)
C21—N3—Cu1	119.8 (2)	C19—C14—C13	117.0 (3)
C20—N4—C24	116.7 (3)	C15—C14—C13	122.5 (3)
C3—O1—Cu1	129.0 (2)	O2—C15—C16	118.9 (3)
C15—O2—Cu1	127.7 (2)	O2—C15—C14	124.2 (3)
N1—C1—C2	125.1 (3)	C16—C15—C14	116.9 (3)
N1—C1—H1	117.5	C17—C16—C15	122.3 (3)
C2—C1—H1	117.5	C17—C16—H16	118.9
C3—C2—C7	119.1 (3)	C15—C16—H16	118.9
C3—C2—C1	123.9 (3)	C16—C17—C18	119.8 (3)
C7—C2—C1	116.6 (3)	C16—C17—H17	120.1
O1—C3—C4	119.2 (3)	C18—C17—H17	120.1
O1—C3—C2	123.6 (3)	C19—C18—C17	120.8 (3)
C4—C3—C2	117.3 (3)	C19—C18—Cl3	119.0 (3)
C5—C4—C3	122.3 (4)	C17—C18—Cl3	120.2 (3)
C5—C4—H4	118.8	C18—C19—C14	119.9 (3)
C3—C4—H4	118.8	C18—C19—H19	120.0
C4—C5—C6	119.9 (3)	C14—C19—H19	120.0
C4—C5—H5	120.0	N4—C20—C21	125.2 (4)
C6—C5—H5	120.0	N4—C20—Cl4	116.0 (3)
C7—C6—C5	120.9 (3)	C21—C20—Cl4	118.8 (3)
C7—C6—Cl1	118.8 (3)	C22—C21—C20	116.6 (3)
C5—C6—Cl1	120.3 (3)	C22—C21—N3	121.7 (3)
C6—C7—C2	120.5 (3)	C20—C21—N3	121.4 (4)
C6—C7—H7	119.8	C21—C22—C23	119.9 (4)
C2—C7—H7	119.8	C21—C22—H22	120.1
N2—C8—C9	125.4 (4)	C23—C22—H22	120.1
N2—C8—Cl2	114.8 (4)	C24—C23—C22	117.9 (4)
C9—C8—Cl2	119.8 (3)	C24—C23—H23	121.0
C10—C9—C8	117.8 (4)	C22—C23—H23	121.0
C10—C9—N1	122.8 (4)	N4—C24—C23	123.6 (4)
C8—C9—N1	119.4 (3)	N4—C24—H24	118.2
C9—C10—C11	118.4 (4)	C23—C24—H24	118.2
			
O1—Cu1—N1—C1	8.0 (3)	C1—N1—C9—C10	53.3 (5)
O2—Cu1—N1—C1	−151.7 (3)	Cu1—N1—C9—C10	−123.5 (3)
N3—Cu1—N1—C1	108.5 (4)	C1—N1—C9—C8	−128.7 (3)
O1—Cu1—N1—C9	−175.6 (3)	Cu1—N1—C9—C8	54.5 (4)
O2—Cu1—N1—C9	24.8 (3)	C8—C9—C10—C11	1.3 (5)
N3—Cu1—N1—C9	−75.0 (4)	N1—C9—C10—C11	179.3 (3)
O1—Cu1—N3—C13	−173.1 (3)	C9—C10—C11—C12	−1.9 (6)
O2—Cu1—N3—C13	−13.5 (3)	C8—N2—C12—C11	−0.6 (7)
N1—Cu1—N3—C13	85.9 (4)	C10—C11—C12—N2	1.7 (8)
O1—Cu1—N3—C21	7.2 (3)	C21—N3—C13—C14	−176.9 (3)
O2—Cu1—N3—C21	166.8 (3)	Cu1—N3—C13—C14	3.4 (5)
N1—Cu1—N3—C21	−93.7 (4)	N3—C13—C14—C19	−175.7 (4)
O2—Cu1—O1—C3	96.9 (4)	N3—C13—C14—C15	8.2 (6)
N1—Cu1—O1—C3	−3.4 (3)	Cu1—O2—C15—C16	167.6 (2)
N3—Cu1—O1—C3	−164.1 (3)	Cu1—O2—C15—C14	−13.5 (5)
O1—Cu1—O2—C15	117.5 (3)	C19—C14—C15—O2	−179.2 (3)
N1—Cu1—O2—C15	−141.7 (3)	C13—C14—C15—O2	−3.3 (6)
N3—Cu1—O2—C15	18.9 (3)	C19—C14—C15—C16	−0.3 (5)
C9—N1—C1—C2	176.8 (3)	C13—C14—C15—C16	175.6 (3)
Cu1—N1—C1—C2	−6.7 (5)	O2—C15—C16—C17	179.5 (3)
N1—C1—C2—C3	−1.7 (5)	C14—C15—C16—C17	0.5 (6)
N1—C1—C2—C7	−175.0 (3)	C15—C16—C17—C18	0.0 (6)
Cu1—O1—C3—C4	176.4 (2)	C16—C17—C18—C19	−0.7 (6)
Cu1—O1—C3—C2	−2.9 (5)	C16—C17—C18—Cl3	−179.8 (3)
C7—C2—C3—O1	−180.0 (3)	C17—C18—C19—C14	0.9 (6)
C1—C2—C3—O1	6.9 (5)	Cl3—C18—C19—C14	180.0 (3)
C7—C2—C3—C4	0.8 (5)	C15—C14—C19—C18	−0.3 (6)
C1—C2—C3—C4	−172.4 (3)	C13—C14—C19—C18	−176.5 (3)
O1—C3—C4—C5	−178.5 (3)	C24—N4—C20—C21	−1.3 (5)
C2—C3—C4—C5	0.8 (5)	C24—N4—C20—Cl4	178.3 (3)
C3—C4—C5—C6	−1.2 (6)	N4—C20—C21—C22	1.5 (5)
C4—C5—C6—C7	−0.1 (6)	Cl4—C20—C21—C22	−178.0 (3)
C4—C5—C6—Cl1	179.1 (3)	N4—C20—C21—N3	−173.4 (3)
C5—C6—C7—C2	1.7 (5)	Cl4—C20—C21—N3	7.1 (4)
Cl1—C6—C7—C2	−177.5 (3)	C13—N3—C21—C22	75.5 (4)
C3—C2—C7—C6	−2.0 (5)	Cu1—N3—C21—C22	−104.8 (3)
C1—C2—C7—C6	171.6 (3)	C13—N3—C21—C20	−109.9 (4)
C12—N2—C8—C9	−0.1 (6)	Cu1—N3—C21—C20	69.8 (4)
C12—N2—C8—Cl2	178.5 (3)	C20—C21—C22—C23	−0.1 (5)
N2—C8—C9—C10	−0.3 (6)	N3—C21—C22—C23	174.8 (3)
Cl2—C8—C9—C10	−178.9 (3)	C21—C22—C23—C24	−1.3 (5)
N2—C8—C9—N1	−178.4 (3)	C20—N4—C24—C23	−0.4 (6)
Cl2—C8—C9—N1	3.0 (4)	C22—C23—C24—N4	1.7 (6)

### Hydrogen-bond geometry (Å, °)

**Table:**

*D*—H···*A*	*D*—H	H···*A*	*D*···*A*	*D*—H···*A*
C22—H22···Cg1^i^	0.93	2.89	3.753 (3)	155

Symmetry codes: (i) *x*, −*y*+3/2, *z*+1/2.
